# An Indoor Accessibility Assessment Framework Based on Multimodal Sensing and Explainable Machine Learning: A Case Study of a Tactile Museum for People with Visual Impairments

**DOI:** 10.3390/s26134198

**Published:** 2026-07-02

**Authors:** Yiqi Tao, Zhiheng Guo, Yusong Zhu, Jingyi Zhang, Zhaohui Yang, Yejin Wang, Yijia Chen, Yuxi Zhou, Fang Liu

**Affiliations:** 1 State Key Laboratory of Subtropical Building and Urban Science, Center for Human-Oriented Environment and Sustainable Design, School of Architecture and Urban Planning, Shenzhen University, Shenzhen 518060, China; taoyiqi@szu.edu.cn (Y.T.); 2310325043@email.szu.edu.cn (Z.G.); 2550111001@mails.szu.edu.cn (J.Z.); yzhaohui2022@163.com (Z.Y.); 2510115031@mails.szu.edu.cn (Y.W.); 2510115042@mails.szu.edu.cn (Y.C.); rainceleste777@gmail.com (Y.Z.); 2 School of Architecture, Southeast University, Nanjing 210096, China; yusong.z@seu.edu.cn

**Keywords:** blind users, multi-sensor data fusion, UWB indoor positioning, indoor accessibility assessment, explainable machine learning, sensor-supported assessment

## Abstract

As accessibility development in public buildings has gradually shifted from facility compliance toward experience- and performance-oriented evaluation, the quantitative assessment of indoor mobility experiences among blind users still lacks a systematic sensor-supported analytical framework. To address this gap, this study proposes an indoor accessibility assessment approach that integrates multi-sensor data acquisition with explainable machine learning, using a tactile museum as the experimental setting. Sixty-four participants with first-level blindness were recruited to complete a real-world directed walking task. A multimodal database was constructed by integrating objective data collected from an ultra-wideband (UWB) indoor positioning system, an intelligent gait analysis system, and video-based behavioral recording, including spatiotemporal trajectories, gait characteristics, and behavioral events, together with post-task accessibility satisfaction ratings. Based on this dataset, a random forest model was developed using the Overall Accessibility Satisfaction Score (OAS) as the response variable. SHAP, partial dependence analysis, and GAM smoothing were further applied to interpret the associations between key variables and predicted satisfaction. The results showed that walking distance, number of turns, self-reported collision perception, and selected gait indicators made relatively high contributions to the model interpretation, and these variables exhibited certain nonlinear associations with predicted satisfaction. These findings suggest that combining multi-source sensor-based behavioral measurement with explainable machine learning has potential for sensor-supported post-occupancy evaluation of indoor accessibility environments and can provide exploratory references for the quantitative assessment and optimization of accessibility in public buildings.

## 1. Introduction

### 1.1. Background

More than 2.2 billion people worldwide have vision impairment or blindness [1]. Their spatial navigation, independent mobility, and access to public services are persistently constrained by environmental conditions. Accordingly, the accessibility, usability, and experiential quality of public buildings and cultural facilities have become important topics in accessibility research [2]. At the international level, the United Nations Convention on the Rights of Persons with Disabilities (UN CRPD) and the European Accessibility Act have promoted the transition of accessibility development from rights advocacy toward institutionalized and standardized implementation. Asian countries such as Japan and South Korea have also improved environmental friendliness for people with visual impairments through legal refinement, wayfinding system optimization, and public space renewal. In China, the number of people with visual impairments exceeded 17 million by 2023 [3], and a regulatory framework has been established based on the Law on the Construction of Accessible Environments and relevant design codes. However, current standards, including the Code for Accessibility Design and the Code for Design of Library Buildings, still provide mainly advisory or principle-based guidance on spatial scale, guidance cues, obstacle control, and circulation organization related to indoor mobility for people with visual impairments. These standards remain insufficient to support refined design and performance evaluation in public cultural buildings [4,5,6,7,8]. In spaces frequently used by people with visual impairments, such as libraries and museums, quantitative evidence based on real behavioral data is still lacking regarding how satisfaction is affected by walking experience, path comprehension, spatial layout, and environmental factors. Therefore, this study focuses on the tactile museum of the China Braille Library. By integrating UWB indoor positioning, gait data acquisition, video-based behavioral coding, and subjective satisfaction-scale data, and by applying random forest and SHAP-based explainable machine learning methods, this study constructs a multi-sensor-data-based evidence-informed spatial assessment framework to provide more quantifiable, interpretable, and translatable parameters for blind-friendly spatial design.

### 1.2. Literature Review

Over the past decade, user-centered design (UCD) research on assistive technologies has developed rapidly in the field of visual impairment. However, existing studies have focused more on system usability validation and outcome-oriented satisfaction measurement, while paying insufficient quantitative attention to the relationship between behavioral performance and subjective experience during real tasks. Ortiz-Escobar et al. conducted a systematic review of relevant studies from 2012 to 2022 based on the ISO 9241-210 framework [9] and found that most studies relied on the System Usability Scale (SUS) or self-developed Likert scales for post-task evaluation, whereas early-stage needs identification, iterative testing, and closed-loop feedback were relatively weak. They therefore recommended that behavioral performance and subjective satisfaction in task-based scenarios be incorporated in parallel into evaluation systems [10]. In cultural building and heritage contexts, Leporini et al. proposed an accessibility design paradigm combining interactive three-dimensional tactile models with audio descriptions, arguing that tools such as 3D printing can help people with visual impairments develop holistic spatial cognition and improve satisfaction and perceived control [11]. In virtual environment research, Zhao et al. showed that multimodal feedback combining haptic damping, vibration, and three-dimensional auditory cues can significantly improve route understanding and wayfinding success among participants with visual impairments [12]. Together, these studies indicate that relying solely on post-task questionnaires is insufficient to reveal the actual benefits of design interventions. Sensor-supported methods capable of continuously recording behavioral processes should therefore be introduced into real or realistic tasks to enable integrated assessment of performance, experience, and feedback.

For first-time visits to unfamiliar indoor environments, navigation usability and user experience depend strongly on the completeness of the location-guidance-feedback system. Guerrero et al. developed and tested an integrated navigation prototype in typical indoor settings such as museums and teaching buildings. Their results showed that the system improved route compliance and perceived usability, while also indicating that some solutions still faced problems such as high deployment costs, complex maintenance, and insufficient compatibility with user habits [13]. Regarding indoor positioning technologies, reviews by Zafari et al. and Leitch et al. compared Wi-Fi, BLE, UWB, and IMU technologies in terms of accuracy, energy consumption, cost, latency, and scalability. They noted that UWB has advantages in positioning accuracy and low latency, whereas BLE is more feasible in terms of cost and deployment flexibility. Multi-source fusion can improve system robustness and reduce the influence of occlusion [14,15]. These studies provide a technical foundation for indoor navigation systems and behavioral sensing. They also suggest that sensors should not be viewed merely as tools for implementing assistive navigation, but can further serve as data entry points for evaluating building-space performance by capturing key behavioral responses such as route choice, stopping, backtracking, and turning in real environments.

Assessing only arrival success or whether users are satisfied is insufficient to explain the actual influence of spatial environments on people with visual impairments; process-oriented indicators must also be introduced. Gait is a key variable connecting environmental load, motor control, and safety risk. Hallemans et al. found that, compared with sighted individuals, people with low vision or blindness show systematic changes in gait speed, step length, and double-support time, reflecting more cautious walking strategies and reduced dynamic stability [16]. Alghadir et al. further confirmed that the postural stability of visually impaired individuals resembles that of sighted individuals under eyes-closed conditions [17]. A systematic review by Kahaki et al. also reported that visually impaired populations generally exhibit reduced walking speed, increased step width, weaker rhythm, and prolonged double-support time [18]. In terms of data acquisition, studies by Reyes-Leiva et al. and Prisco et al. demonstrated that wearable IMUs and their integration with vision-based methods can effectively estimate key spatiotemporal gait parameters, supporting field data collection with higher ecological validity [19,20]. These findings suggest that building-space assessment should not be limited to static dimensional review or post-task questionnaires. Instead, a sensor-driven multi-layer data acquisition framework should be used to combine gait parameters, trajectory features, and event-based behavioral coding to identify potential obstacles and risk triggers from the perspective of dynamic behavior.

However, the relationships among spatial characteristics, behavioral processes, and subjective satisfaction are often not simply linear. Threshold effects and interactions may also exist among different factors, which traditional linear statistical methods are unable to fully reveal. In recent years, machine learning and explainable artificial intelligence methods have been widely applied to studies modeling environment-experience relationships. Lee et al. used random forest (RF) and XGBoost with SHAP explanations based on streetscape visual and environmental characteristics, identifying the thresholds and interaction effects of variables such as sidewalk proportion, greenery, and street facilities on pedestrian satisfaction [21]. Paul et al. integrated questionnaire and sensor data and used LightGBM and RF models to reveal the differential impacts of vehicle distance, traffic noise, and pedestrian density across travel contexts [22]. Methodologically, the random forest algorithm proposed by Breiman can effectively handle nonlinear relationships, high-dimensional inputs, and heteroscedasticity [23]. The SHAP method proposed by Lundberg and Lee can be used to explain the direction and magnitude of variable contributions and local prediction differences [24]. In addition, to obtain a smoother and more interpretable feature value-effect relationship, this study introduces a generalized additive model (GAM) to fit the SHAP results, following related applications by Jia et al. [25,26]. The feature value is used as the independent variable, and the corresponding SHAP value is used as the dependent variable to establish a smooth function. The location where the SHAP value equals zero is regarded as the critical point at which the influence direction of the feature changes. In this way, sensor acquisition is no longer limited to raw data recording but is embedded in an assessment chain from recording and modeling to interpretation, allowing behavioral sensing results to support building-space diagnosis and optimization.

Overall, existing research has accumulated foundations in assistive technology design, indoor navigation systems, gait-risk characterization, and explainable modeling. Nevertheless, studies remain limited in integrating multimodal sensing data, behavioral-process indicators, and subjective satisfaction evaluation into a unified framework in real public building settings. Accordingly, this study uses the tactile museum of the China Braille Library as experimental settings to construct a multimodal sensing framework for accessibility assessment in building spaces. OptoGait data, UWB trajectory data, and video-coded behavioral data are jointly collected during real tasks and aligned with satisfaction-scale responses. Random forest modeling combined with explainable machine learning is then used to explore nonlinear relationships between features such as obstacle-related behaviors and gait parameters and satisfaction. Compared with traditional evaluation approaches based primarily on static code checking or single questionnaires, the contribution of this study lies in combining sensor-driven behavioral data acquisition, explainable machine learning, and building-space assessment, thereby providing a reusable sensor-enabled assessment framework for diagnosing accessibility performance in public buildings.

## 2. Methodology

### 2.1. Human-Factor Data Collection

**Figure 1:**
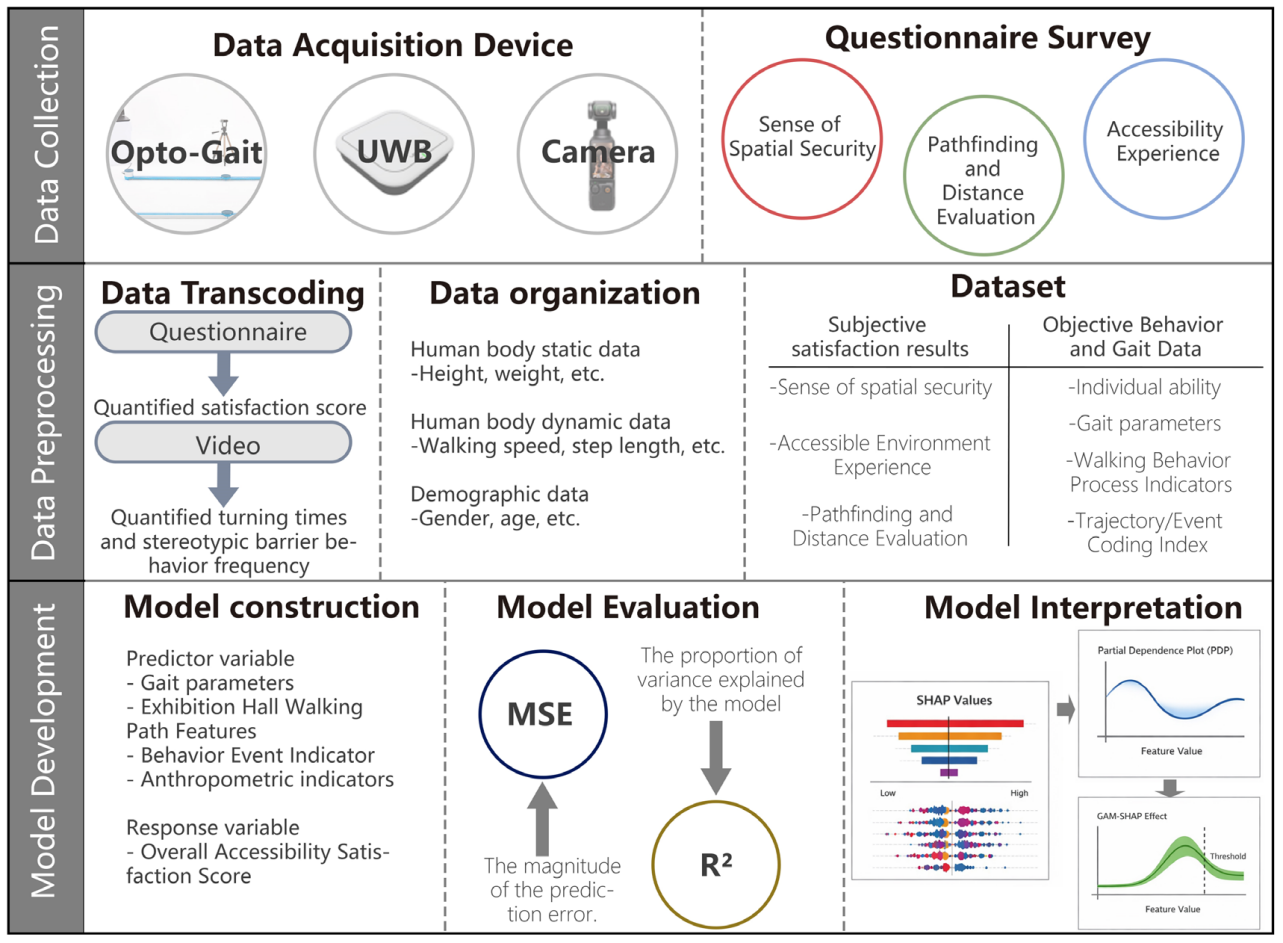
Research framework.

Figure 1 presents the overall workflow of the proposed human-factors data collection and analysis framework. The framework consists of three sequential stages: data collection, data preprocessing, and model development. In the data collection stage, multimodal human-factors data were obtained from both objective sensing devices and subjective questionnaire surveys. Specifically, OptoGait (Microgate S.r.l., Bolzano, Italy) was used to record participants’ gait parameters, UWB positioning was used to obtain walking distance and walking time during the exhibition-hall task, and video cameras were used to support subsequent behavioral event coding, including turning and stereotyped barrier-related behaviors. Meanwhile, questionnaire surveys were conducted to measure participants’ perceived spatial security, pathfinding and distance evaluation, and accessibility experience. These subjective and objective data were then transcribed, organized, and integrated into a unified dataset, including subjective satisfaction scores, individual characteristics, gait parameters, walking behavior process indicators, and trajectory/event coding indices. The integrated dataset provided the basis for the subsequent random forest modeling, model evaluation, and interpretation analyses.

#### 2.1.1. Architectural Space Information and Participants

**Figure 2:**
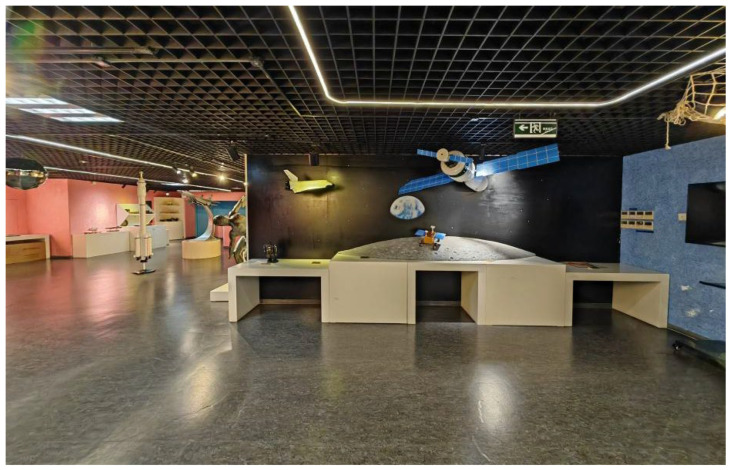
Photographs of the exhibition hall.

The experimental site for walking-behavior data collection was the tactile museum for people with visual impairments affiliated with the China Braille Library, while gait data were collected in a nearby quiet room free from interference. The tactile museum is a typical public indoor space open to people with visual impairments (Figure 2). It displays various exhibits that visitors can touch. The museum has a moderate floor area and a viewing route with a certain degree of complexity; therefore, it was considered representative for this experiment.

The study included 64 participants with first-level blindness, classified according to the Chinese standard GB/T 26341-2010 [27] and verified on site using each participant’s People’s Republic of China Disability Certificate. Participants ranged in age from 19 to 73 years, including 35 males and 29 females. This sample size is comparable to previous studies [28] and is larger than that in most studies recruiting real participants with visual impairments [29,30,31]. Apart from visual impairment, the recruited participants had no other registered disabilities. All participants were able to walk independently and clearly understand the experimental instructions, and none had other diseases that would significantly affect the experiment. Participants were required to have sufficient sleep, refrain from alcohol consumption, and avoid psychotropic drugs. This study was approved by the Medical Ethics Committee of Shenzhen University (PN-202500123).

#### 2.1.2. Gait Data Measurement

**Figure 3:**
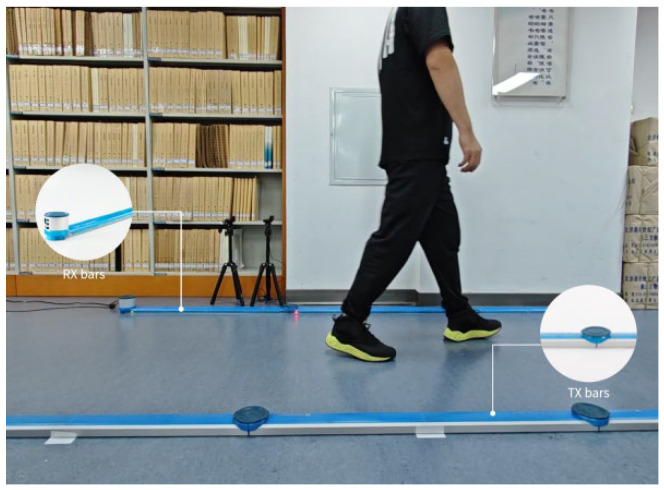
OptoGait system and gait measurement setting.

This study used the OptoGait photoelectric gait system to collect spatiotemporal gait parameters from the participants (Figure 3). OptoGait consists of oppositely arranged transmitting and receiving photoelectric bars, with dense diode matrices installed on the bars. When the foot passes through the sensing area, the system records foot-contact and toe-off events, from which multiple spatiotemporal gait parameters can be calculated. Previous studies have confirmed that OptoGait shows good agreement and reliability compared with electronic walkways, such as GAIT Rite, and three-dimensional motion-capture systems [32,33,34], making it suitable for gait measurement in clinical and research settings. At the beginning of the test, participants stood in the waiting area and faced a distant acoustic beacon to identify the walking direction. After the staff gave an auditory prompt, participants walked straight through the sensing area at their preferred comfortable speed. To reduce unfamiliarity and nervousness during the first attempt, three familiarization trials were arranged before formal data collection. During formal data collection, each participant completed at least two valid one-way passes to obtain steady-state walking data [35,36,37]. Finally, valid gait parameters exported from the OptoGait analysis software were used for subsequent analysis. The gait indicators included pre-test mean gait speed, minimum gait speed, maximum gait speed, step length, step frequency, gait cycle duration, stance phase and its percentage, swing phase and its percentage, step time, and left–right step time asymmetry. These indicators were used to characterize participants’ basic motor capacity, spatiotemporal gait features, and movement stability under unobstructed straight-line walking conditions.

#### 2.1.3. Walking Behavior Data Measurement

**Figure 4:**
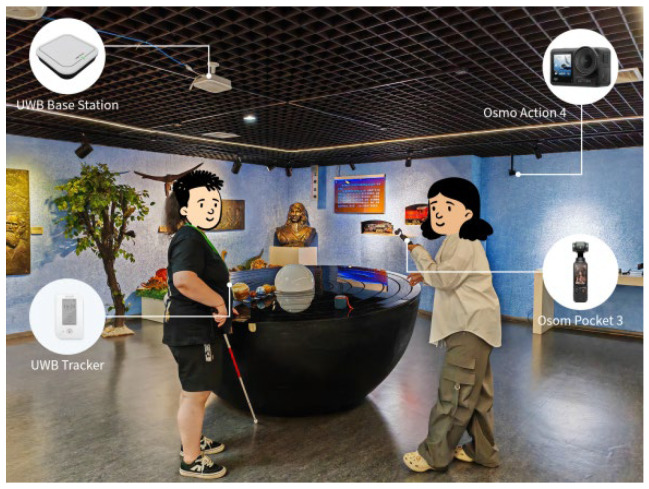
Multimodal data-collection setup in the tactile museum.

**Figure 5:**
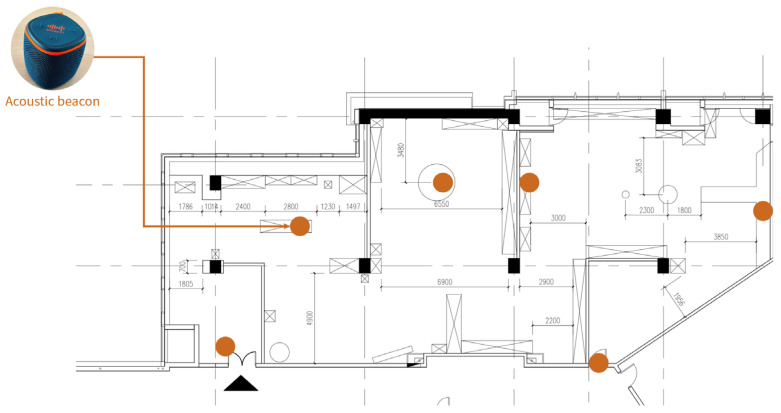
Schematic diagram of check-in points in the experimental setting.

To stably obtain walking-behavior characteristics under an approximate “independent exhibition viewing” scenario, this study adopted a wayfinding task (Figure 4). Participants followed acoustic beacons to visit six tactile check-in points in sequence and finally arrived at the exit (Figure 5; six acoustic beacons were installed in the venue, corresponding to six check-in points in the scene). Before the experiment began, participants prepared in the lounge outside the tactile museum. After receiving instructions from the experimenter, participants started walking independently by following the beacons, visited the six points in sequence, and touched the corresponding beacons. Referring to previous studies involving auditory beacons, this paradigm can elicit collision, stopping, and avoidance behaviors without interfering with the exhibition layout, and has been shown to effectively trigger target-oriented localization responses in indoor navigation and accessible auditory-guidance studies [38,39,40].

**Figure 6:**
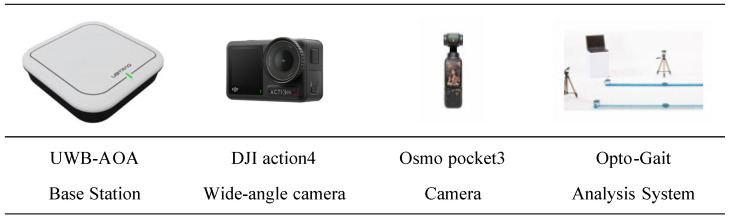
Key experimental equipment.

For positioning data collection, an Ultra-Wideband (UWB) indoor positioning module was used (Figure 6). The positioning network consisted of ceiling-mounted anchors and participant-worn tags (Figure 4). UWB has relatively high positioning accuracy and temporal stability in complex indoor environments, making it suitable for personnel trajectory collection and flow analysis or guidance applications in museum-like spaces [41,42,43,44]. In this study, the UWB indoor positioning module was used to obtain route-level kinematic indicators, including walking distance, walking duration, and mean task speed. The UWB positioning system consisted of positioning anchors, wearable tags, and backend trajectory-recording software. Before the experiment, the researchers placed a lightweight UWB positioning tag on the participant’s upper trunk to reduce the influence of arm swing and white-cane use on trajectory recording. Eight UWB anchors were installed under the ceiling at an approximate height of 2.4 m above the floor to cover the main walking route and turning nodes. Before the experiment, the researchers checked the signal quality between the tag and the anchors through the system backend and confirmed that the trajectory-recording interface could stably display the participant’s position. The UWB device model was UT100-AM, with a sampling rate of 8 Hz. According to the China Academy of Information and Communications Technology test report, the maximum planar positioning error of this device model under standard testing conditions is less than 20 cm.

Detailed behaviors were obtained through video-based observation and coding. Fixed cameras were placed in the exhibition area to cover key turns and congested nodes, while a handheld camera followed participants to record close-up movement details (Figure 4). The coding ontology included collision/contact (visible contact with exhibits, walls, or other people), getting lost/deviation (deviation from the planned route or missed beacons followed by detouring, asking for help, or obvious hesitation lasting at least 3 s), turning (defined as a turn angle of at least 45° based on body orientation and movement direction), and optional stopping (continuous stillness or near-stillness lasting at least 2 s). These definitions were developed with reference to video-behavior studies in museum and indoor scenes and threshold evidence from pedestrian-turning experiments; a 45° turning threshold can effectively distinguish the influence of turning angles on walking behavior [44,45]. Behavioral-event coding was completed using an on-site multi-person consensus procedure. Before the experiment, researchers provided unified explanations of the predefined behavioral definitions to the coders and clarified the criteria for collision, getting lost/deviation, turning, and stopping using pilot procedures. During formal experiments, five researchers simultaneously observed each participant’s walking process on site and recorded behavioral events by raising cards according to the predefined criteria. When different coders disagreed on an event, the final event type was determined by majority agreement. Fixed-camera and handheld videos were also recorded throughout the experiment to document the process and, when necessary, assist in checking the event context. Questionnaire scoring was conducted separately after the walking task; therefore, behavioral coding was independent of questionnaire responses, reducing the influence of questionnaire evaluation on event recording.

#### 2.1.4. Data Preprocessing and Indicator Calculation

UWB trajectory data were first segmented according to the task start and end times, retaining only the trajectory records from the moment participants began the directed walking task to the moment they reached the destination. The task start time was defined as the time when the experimenter gave the start command and simultaneously initiated UWB trajectory recording and video capture. The end time was defined as the time when the participant reached the exit and the recording equipment was stopped. Trajectory organization and distance calculation were performed in ArcGIS10.5 and Python3.9. Walking distance was calculated based on the planar relative coordinates output by the UWB system and was obtained by accumulating the planar Euclidean distances between adjacent trajectory points:

(1) $$D=\sum_{i=2}^{n}\sqrt{(x_{i}-x_{i-1}{)}^{2}+(y_{i}-y_{i-1}{)}^{2}}$$

D = Σ√[(x_i − x_{i−1})^2^ + (y_i − y_{i−1})^2^]

where x_i and y_i denote the planar coordinates of the i-th valid UWB trajectory point. To ensure consistency between the trajectory data and the actual task process, the task start and end times were checked again against the video records. Mean task speed was calculated by dividing walking distance by walking duration:

(2) $$V=\frac{D}{T}$$

where D denotes walking distance and T denotes walking duration. It should be noted that this study did not perform complex interpolation correction or micro-trajectory reconstruction on the UWB trajectories. Given that the UWB sampling rate provided sufficient temporal resolution relative to the walking speed in this study, and because the analysis focused on route-level overall kinematic indicators, such as total walking distance, total walking duration, and mean speed, UWB trajectory data were mainly used to reflect the overall path burden during the task. Event-level behaviors, such as turning, collision/contact, and getting lost, were not directly identified from UWB trajectories; instead, they were obtained through video coding to avoid event-recognition bias caused by local positioning errors or short-term occlusions.

#### 2.1.5. Questionnaire Surveys

To obtain participants’ subjective accessibility experience after walking in the exhibition hall, this study administered a self-developed questionnaire immediately after the experiment. The questionnaire included three dimensions: perceived spatial safety, accessible-environment experience, and wayfinding and distance evaluation. All items used a five-point Likert scale and were contextualized according to the actual exhibition-hall environment, covering object recognition, aisle width, route-finding ease, distance between exhibition booths, entrance passability, and exit guidance. The complete questionnaire items, English translations, dimensional affiliations, scoring directions, and whether each item was included in the Overall Accessibility Satisfaction Score (OAS) are provided in Appendix Table A3.

To examine the internal consistency of the questionnaire, Cronbach’s alpha and McDonald’s omega were jointly used for reliability analysis. After excluding open-ended items and navigation-preference items, the complete questionnaire showed high internal consistency, with Cronbach’s alpha = 0.836 and McDonald’s omega = 0.845, indicating that the questionnaire as a whole could relatively consistently reflect participants’ accessibility-experience evaluations.

The Overall Accessibility Satisfaction Score (OAS) was calculated by summing seven post-task evaluation items and was used to represent participants’ overall evaluation of the accessibility experience in the experimental space. All items included in the OAS were coded in the same evaluative direction, with higher scores indicating higher overall accessibility satisfaction. The theoretical range of the OAS was 7–35. For the seven OAS items, Cronbach’s alpha was 0.525 and McDonald’s omega was 0.709. The relatively low Cronbach’s alpha may be related to the multidimensionality of the OAS items, as these items involved several related but not identical aspects of experience, such as spatial safety, route understanding, distance evaluation, and entrance/exit guidance. In contrast, McDonald’s omega provides a more appropriate estimate of internal consistency when item contributions are not fully equivalent, and the result reached an acceptable level.

To further examine the consistency between the OAS component items and the total score, the corrected item-total correlation (CITC) of the seven OAS items was calculated. The results showed that all seven items had positive CITC values, ranging from 0.098 to 0.361, indicating that the items were generally consistent with the overall accessibility-experience evaluation. Detailed results will be provided as Supplementary Material. At the same time, some items showed relatively low CITC values, suggesting that the OAS is not a strictly unidimensional psychometric scale, but rather an integrated post-task evaluation indicator covering multiple experiential aspects, such as safety perception and route understanding. This characteristic is common in applied studies focusing on environmental experience and satisfaction [46,47,48]; therefore, this study used the OAS as an exploratory composite response variable for subsequent modeling. In addition, self-developed scales are commonly used in environmental psychology, accessibility research, and post-occupancy evaluation (POE), particularly when existing standardized tools cannot fully cover a specific context or population. In recent years, several studies have used self-developed items to measure perceived residential environments and environmental satisfaction [49,50], and other studies have used self-developed questionnaires to evaluate environmental quality and satisfaction in long-term care facilities [51]. These studies provide methodological support for the use of a self-developed questionnaire in the specific spatial context of this study.

### 2.2. Response and Predictor Variables

To avoid conflating variables from different sources during interpretation, this study further classified the predictors according to data source. The response and predictor variables are shown in Appendix Table A1 and Table A2. Predictors included sensor-derived variables, video-coded behavioral variables, subjective questionnaire variables, and demographic/anthropometric variables. Among them, walking distance, walking duration, and mean real-space task speed obtained from UWB positioning, as well as gait parameters obtained from OptoGait, were classified as sensor-derived variables. The number of obstacle-related behaviors, number of turns, and turning redundancy index (Tux) were classified as video-coded behavioral variables. Self-reported collision frequency and self-reported turning frequency were classified as post-task questionnaire variables. These questionnaire variables were not treated as objective behavioral evidence directly generated by sensors, but rather as part of participants’ perceived evaluations.

### 2.3. Machine Learning Algorithm (Random Forest, RF)

This study used Random Forest (RF) regression to model the dataset. The RF model was implemented using the open-source machine-learning library scikit-learn. The dataset was split into training and test sets at an 80%/20% ratio: the training set was used for model fitting, and the test set was used for performance evaluation. The key model parameters were set as n_estimators = 100 (number of trees) and random_state = 42 (random seed). For the regression task, the coefficient of determination (R^2^) and mean squared error (MSE) were used as the main evaluation metrics. To reduce the influence of a single train-test split on model-performance evaluation, this study further adopted repeated 5-fold cross-validation to examine model stability. Specifically, the sample was divided into five folds, and cross-validation was repeated 20 times, producing 100 validation results in total. The mean and standard deviation of R^2^ and MSE were then reported to describe the performance fluctuations of the model under different data partitions. The original fixed 80/20 split was primarily used for subsequent SHAP, PDP, and GAM-SHAP interpretation analyses, while the repeated cross-validation results were used to evaluate the robustness of model performance. An R^2^ value closer to 1 indicates a higher degree of model explanation for the target variable; the MSE ranges from 0 to +∞, and smaller values indicate lower prediction error. Considering the scale of the target variable and the interpretive needs of the study, R^2^ and MSE were used to report model performance.

### 2.4. Model Explanation and Interpretability Analysis

Random forest has advantages in prediction performance and robustness, but its internal decision-making mechanism lacks interpretability and is often considered a “black-box” model. To improve interpretability and support sensitive-change-point identification and design translation, this study constructed an explanatory framework centered on SHAP, combining global-importance visualization, marginal-effect curves, and smooth fitting to interpret model outputs at multiple levels.

First, SHAP (SHapley Additive exPlanations) was used to explain model predictions. Based on the Shapley value in cooperative game theory, SHAP quantifies the marginal contribution of each feature to a prediction as a SHAP value. For an input sample x, the explanation model can be written in additive form as:

(3) $$g\left(x^{\prime }\right)=\phi_{0}+\sum_{i=1}^{M}\phi_{i}x_{i}^{\prime }$$

where g is the explanation model; z’ is the simplified binary representation (1 indicates that a feature is included/present, and 0 indicates that it is missing/not included); M is the number of features; φᵢ is the contribution of feature i to the prediction of that sample, namely the SHAP value; and φ_0_ is the baseline term.

In the classical Shapley framework, the contribution of the i-th feature for sample x can be expressed as the weighted average of its marginal contributions across all possible feature subsets S that do not include i:

(4) $$\phi_{i}=\sum_{S\subseteq F\setminus \{i\}}\frac{|S|!(|F|-|S|-1)!}{|F|!}\left(f_{S\cup \left(i\right)}\left(x_{S\cup \left(i\right)}\right)-f_{S}\left(x_{S}\right)\right)$$

where F is the set of all features, S is any subset that does not contain feature i, f_S denotes the model output trained or estimated using only subset S, and x_S denotes the values of sample x on feature subset S. The weighting term balances the comparability of marginal contributions across subsets of different sizes.

This study used SHAP to rank the importance of features influencing indoor accessibility satisfaction in the experimental scene, thereby providing a basis for subsequent analysis. To further characterize model response relationships for key features, Partial Dependence Plots (PDPs) were used as a global interpretability supplement. By showing the marginal change in model prediction when a given feature varies while the distribution of other features is held constant, PDPs are suitable for identifying potential sensitive ranges and plateau effects, and they complement SHAP results in terms of feature importance and directionality.

Building on the preceding interpretability analyses, this study further used a generalized additive model (GAM) to fit SHAP values. Referring to the approach used by Jia et al. [26], feature values were treated as independent variables and the corresponding SHAP values as dependent variables for smoothing. A SHAP value of 0 was regarded as the point at which the direction of a feature’s contribution to the model prediction changed: when the SHAP value of a feature was greater than 0, the feature contributed positively to the predicted accessibility-satisfaction score at that value; when the SHAP value was less than 0, the feature contributed negatively by lowering the predicted value. This process was used to obtain the overall trend, turning points, and rate of change in the influence of each feature on satisfaction scores, providing a basis for sensitive-change-point identification and spatial-optimization suggestions. It should be noted that SHAP, PDP, and GAM-SHAP analyses were used to explain the prediction patterns of the random forest model in the current sample. They can reveal nonlinear associations between variables and predicted satisfaction, but they cannot demonstrate causal effects of variables on accessibility satisfaction. Because variables such as walking distance, number of turns, deviation, collision/contact, and stopping may be jointly influenced by the same route structure and spatial layout, PDP results may be affected by correlated-feature bias. Therefore, this study interprets PDP and GAM-SHAP results as exploratory model associations and sensitive change points, rather than validated causal thresholds or universal spatial-optimization parameters.

## 3. Experimental Procedure

The experimental procedure followed a fixed five-step sequence, and all 64 participants completed all stages during a single visit. After participants arrived at the China Braille Library, they were first received by an experimenter in a quiet reception area. The experimenter introduced the research purpose and overall procedure in an accessible manner and assisted in obtaining informed consent according to the protocol approved by the Medical Ethics Committee of Shenzhen University (PN-202500123). Basic participant information was then recorded.

After the initial registration, participants completed measurements of height, body weight, and limb lengths in a quiet room. Wearing flat shoes, they then walked along the OptoGait photoelectric walkway at their self-selected comfortable speed. Before formal data collection, the experimenter verbally explained the layout and demonstrated the start and end positions using a buzzer. Participants first completed several familiarization trials, with the experimenter standing nearby to prevent collisions. The OptoGait system automatically recorded parameters such as gait speed, step length, step frequency, gait cycle, swing-phase percentage, and left-right gait-time symmetry. After disturbed trials were excluded, the average of valid trials was used as the gait indicator.

**Figure 7:**
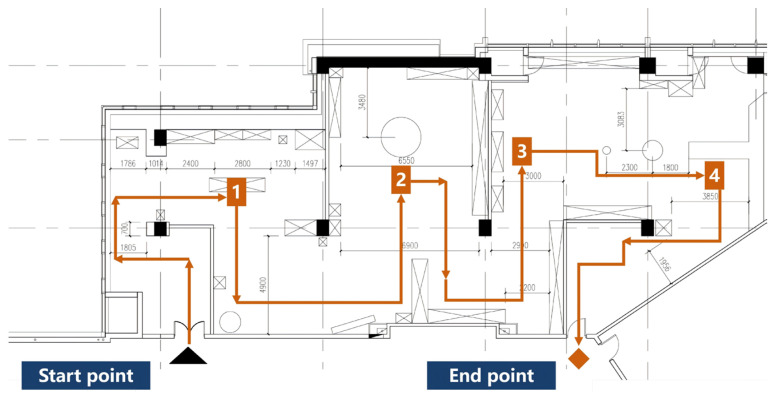
Schematic diagram of the ideal walking route(Numbers 1–4 denote different check-in points respectively).

**Figure 8:**
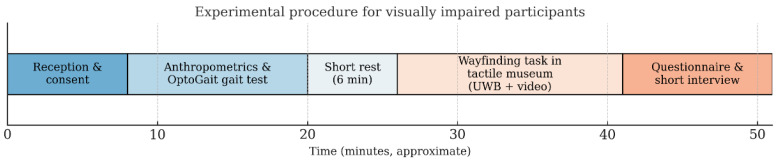
Experimental procedure diagram.

After gait data collection, participants rested for 6 min and then entered the tactile museum. A UWB tag was attached to the participant’s upper trunk, and the experimenter guided the participant to the starting point and provided standardized instructions: the participant was to independently complete a “find–touch–continue” tour task using acoustic beacons. The experimenter did not provide route guidance and intervened only when there was a safety risk. UWB trajectory recording and video capture were started simultaneously, using fixed cameras and handheld following shots (Figure 4). Participants then began wayfinding in a mode that was “free exploration with explicit targets.” Each participant independently completed the full route from the lounge entrance to the exhibition-hall exit (Figure 7); only one participant walked at a time to avoid interference. After reaching the exit, all recordings were stopped, the UWB tag was removed, and participants returned to the reception area, where the experimenter read each questionnaire item aloud and recorded the responses. The overall experimental duration for each participant was approximately 35–50 min (Figure 8).

## 4. Results

### 4.1. Random Forest Model Construction and Performance Evaluation

The model-training results showed that the model had a certain degree of exploratory explanatory ability for overall accessibility satisfaction. Under the fixed random seed (random_state = 42) and the 80%/20% train-test split, the random forest model achieved an R^2^ of 0.424 and an MSE of 8.202. Considering the limited sample size of this study and the potential influence of random data partitioning on a single train-test split, repeated 5-fold cross-validation was further used to examine model robustness. The results showed that after 20 repetitions of 5-fold cross-validation, the mean R^2^ was 0.170 ± 0.325 and the mean MSE was 5.378 ± 1.809. These results indicate that the model showed moderate explanatory ability under the single fixed split, but its prediction stability still fluctuated under small-sample conditions. Therefore, this study does not treat the random forest model as a highly stable predictive model, but rather as an exploratory explanatory tool for identifying potential factors associated with accessibility satisfaction. The subsequent SHAP, PDP, and GAM-SHAP results are all interpreted as model-association patterns under the current sample and scene conditions, rather than stable predictive evidence or generalizable design thresholds.

### 4.2. Feature Importance Ranking and Global Interpretation Based on SHAP Values

To reveal the relative contribution and direction of different features in the random forest model’s prediction of overall accessibility satisfaction, this study used SHAP to interpret the trained model and plotted SHAP bar-summary and beeswarm plots to visualize variable importance and global-effect patterns.

**Figure 9:**
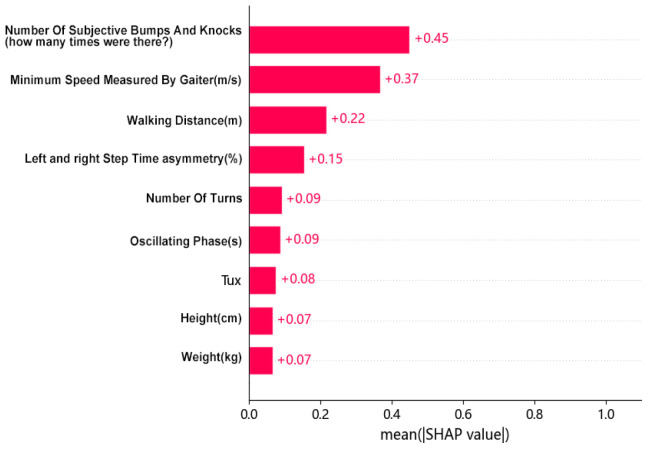
SHAP feature-importance ranking.

The SHAP bar-summary plot (Figure 9) shows that model prediction was mainly driven by a small number of key features. Using mean (|SHAP value|) to measure global contribution strength, self-reported collision frequency had the highest contribution among all variables (approximately 0.45), followed by minimum gait speed measured by the gait analyzer (approximately 0.37), walking distance (approximately 0.22), and left–right step time asymmetry (approximately 0.15). The number of turns, swing-phase percentage, Tux index, height, and body weight contributed sequentially less. Overall, this ranking indicates that, compared with purely demographic characteristics, features that directly reflect walking safety events, path cost, and gait stability provided stronger explanatory information for satisfaction; other variables also participated in the explanation, but their individual effects were relatively limited.

**Figure 10:**
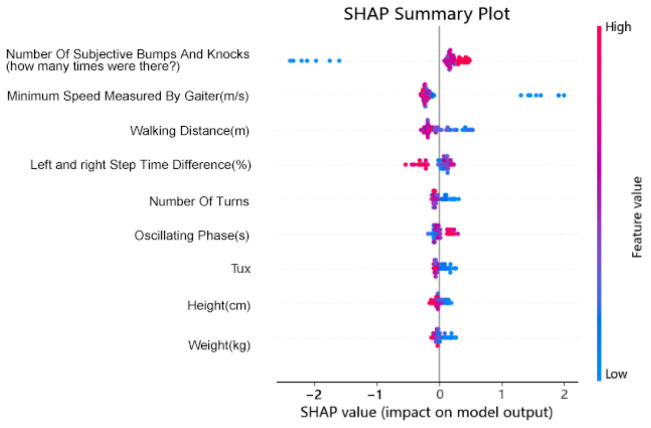
SHAP beeswarm plot showing the global contribution of each feature to the Overall Accessibility Satisfaction Score.

The SHAP bar-summary plot provides a quantitative assessment of each feature’s explanatory contribution in the current model. Furthermore, the beeswarm plot was used to explore the positive and negative effects of the top nine key features on model prediction, as shown in Figure 10. For “self-reported collision frequency,” the feature with the highest predictive contribution in the model, high values were more often located on the positive SHAP side, whereas low values tended to be located in the negative region. In the data coding, high values corresponded to “fewer collisions and better collision conditions,” while low values corresponded to “more collisions and poorer collision conditions.” Self-reported collision frequency was derived from the post-task questionnaire and reflected participants’ subjective perception of collisions rather than objective collision counts directly obtained from sensors or video coding. Therefore, this result should be interpreted as an association between subjective safety perception and overall accessibility satisfaction, rather than as an independent predictive effect of sensor-derived variables on satisfaction. For variables such as walking distance, left–right step time asymmetry, number of turns, Tux index, and body weight, high values (red) were mostly concentrated in the negative SHAP region, while low values (blue) were more often distributed to the right of zero. This indicates that individuals with longer walking routes, more asymmetric gait, more frequent turning, poorer overall gait stability, or higher body weight were more likely to have lower predicted accessibility satisfaction.

Minimum gait speed showed a different trend: higher minimum speed corresponded to negative SHAP values. This suggests that, in the indoor exhibition-hall context of this study, maintaining a faster walking strategy did not necessarily lead to higher satisfaction and may instead have been associated with experiences such as tense avoidance or increased operational burden under spatial constraints. For swing phase, the beeswarm plot showed that high-value samples were still partly located to the right of zero, suggesting that higher swing-phase values contributed positively to satisfaction to some extent.

### 4.3. Marginal Effect Analysis Using One-Dimensional Partial Dependence Plots

In this section, based on the key influencing variables identified in Section 4.2, one-way Partial Dependence Plots (PDPs) were generated for the top six key variables to examine the marginal-effect curves of changes in a single variable on predicted accessibility satisfaction while averaging over the observed distribution of the other features.

**Figure 11:**
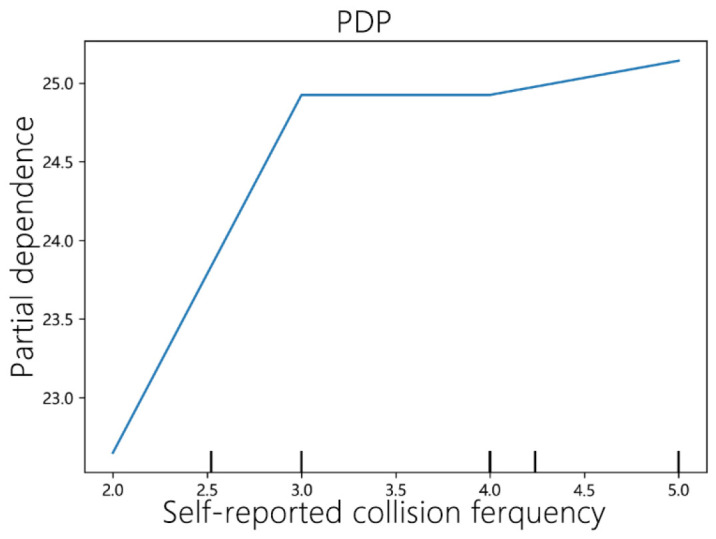
Average effect of Self-reported collision frequency on predicted satisfaction.

**Figure 12:**
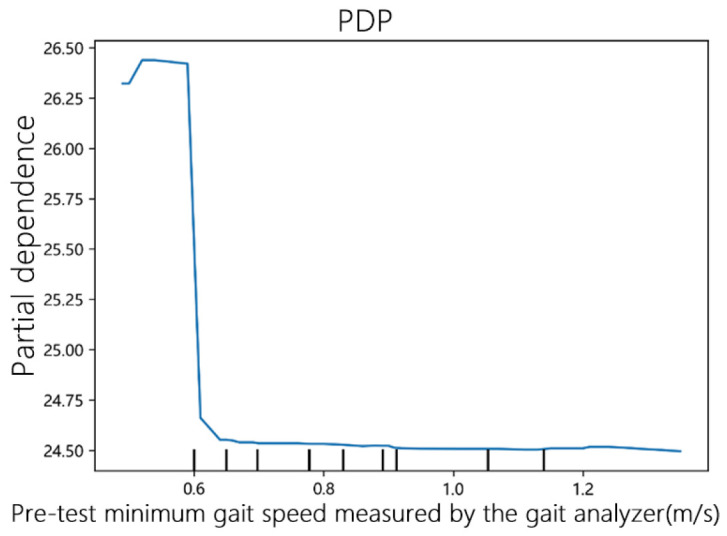
Average effect of Pre-test minimum gait speed measured by the gait analyzer on predicted satisfaction.

**Figure 13:**
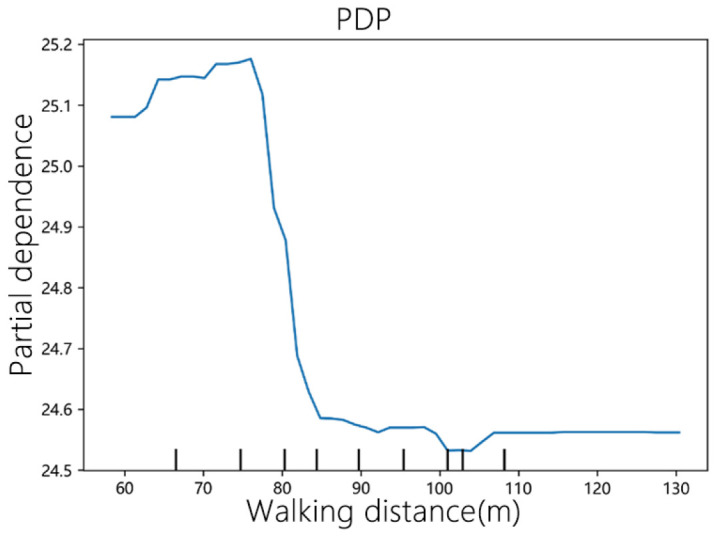
Average effect of Walking distance on predicted satisfaction.

**Figure 14:**
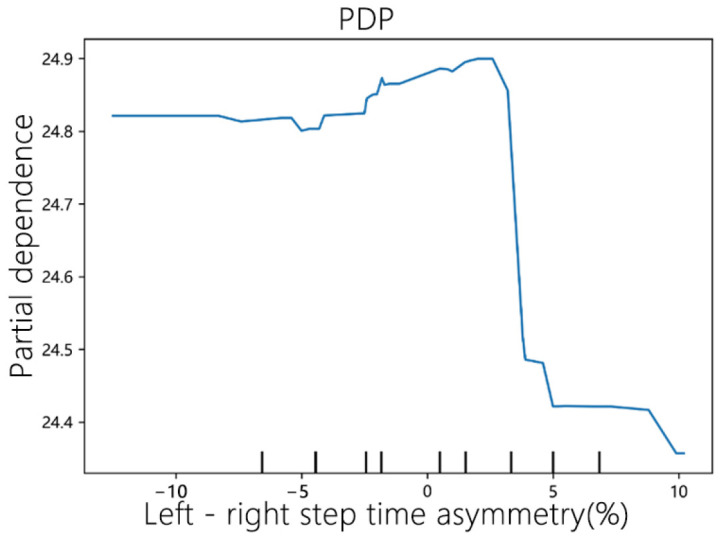
Average effect of Left–right step time asymmetry on predicted satisfaction.

**Figure 15:**
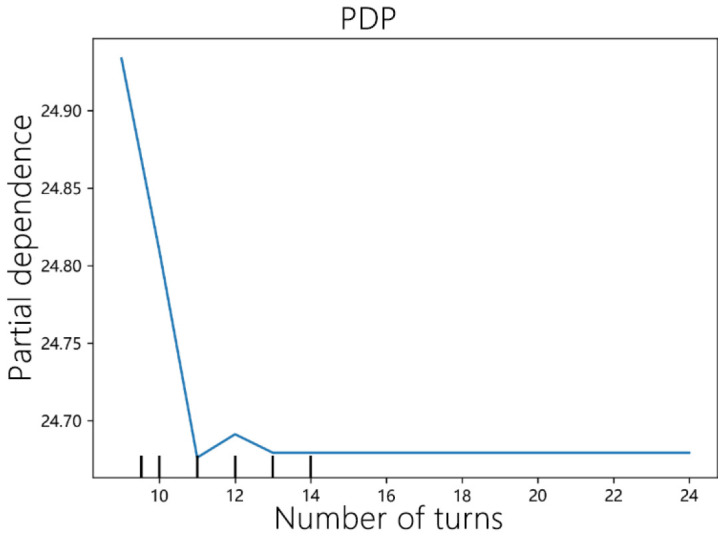
Average effect of Number of turns on predicted satisfaction.

**Figure 16:**
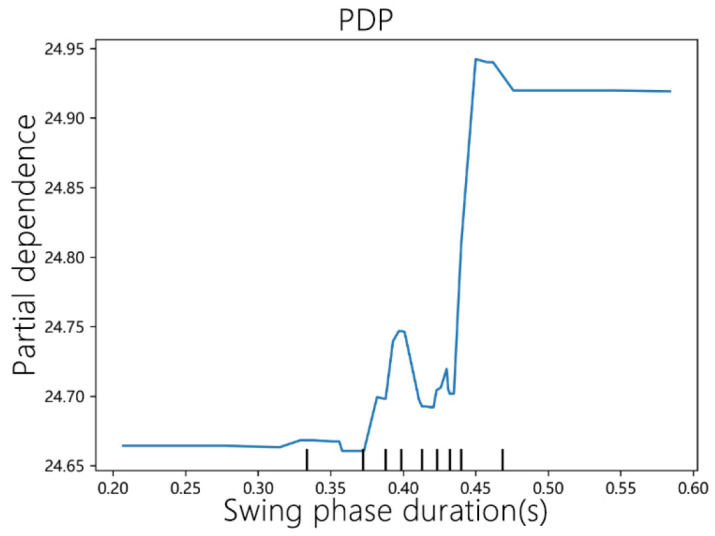
Average effect of Swing phase duration on predicted satisfaction.

In the one-way PDPs (Figure 11, Figure 12, Figure 13, Figure 14, Figure 15 and Figure 16), the vertical axis represents the accessibility satisfaction predicted by the random forest model, while the horizontal axis represents the value of a single feature. The marginal effect of each indicator was estimated by averaging model predictions over the observed distribution of the remaining features. The results showed that as the score for self-reported collision frequency increased (indicating that the participant subjectively perceived fewer collisions), the predicted satisfaction increased from below approximately 23 points to above 25 points. After the score reached around 3 points, the change in satisfaction became less pronounced (Figure 11). This suggests that moderately reducing subjective collision burden can bring substantial benefits, but further improvement has limited marginal effects. The horizontal-axis values represent participants’ subjective perception rather than actual collision counts.

In Figure 12, a turning point appeared at a minimum gait speed of approximately 0.6 m/s, as measured by the gait analyzer. When the minimum speed was relatively low, model-predicted satisfaction remained above 26.25 points; once the minimum speed exceeded 0.6 m/s, it dropped rapidly and remained at a low level thereafter. Walking distance showed a slight positive increase within the range of 60–76.4 m and remained at the highest level across the entire curve. After walking distance exceeded 77 m, the curve declined rapidly and tended toward a low plateau after 84 m (Figure 13). This indicates that a moderate walking distance may help participants complete the task and become familiar with the environment, whereas an overly long path is often associated with detouring or getting lost and corresponds to lower satisfaction.

Regarding gait dynamics and individual characteristics, the figure shows that left–right step time asymmetry had a limited effect on satisfaction within the range of −10% to 3.2%, showing a slight positive effect, but declined steeply between approximately 3.2% and 5%, after which it remained at a low level (Figure 14).

The number of turns corresponded to the highest satisfaction around 9–10 turns, then decreased rapidly between 10 and 11 turns and remained low after 13 turns, with no obvious further change. This suggests that excessive turning significantly increases spatial cognitive load (Figure 15). Swing-phase duration showed a slight increase in satisfaction when it varied from 0.2 to 0.41 s, followed by a marked increase between 0.43 and 0.46 s, reaching a peak around 0.46 s and then slightly decreasing while remaining at a high level. This suggests that a slightly longer and more relaxed swing phase was associated with a better accessibility experience (Figure 16).

Overall, the six PDPs jointly show nonlinear associations and possible trends between key indicators and model-predicted satisfaction, providing exploratory references for spatial optimization and guidance strategies in similar settings. However, PDP results are derived from the current sample and model settings, and variables such as walking distance, number of turns, deviation, and collision/contact may be jointly influenced by the same route and spatial layout. Therefore, these trends should not be interpreted as validated general design thresholds.

### 4.4. Nonlinear Main Effects and Turning-Point Identification Based on GAM-SHAP

**Figure 17:**
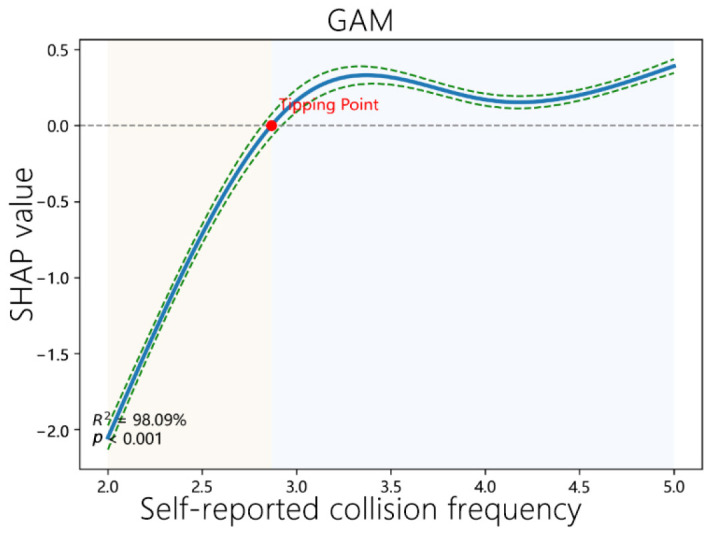
Nonlinear relationship between Self-reported collision frequency and SHAP value.

**Figure 18:**
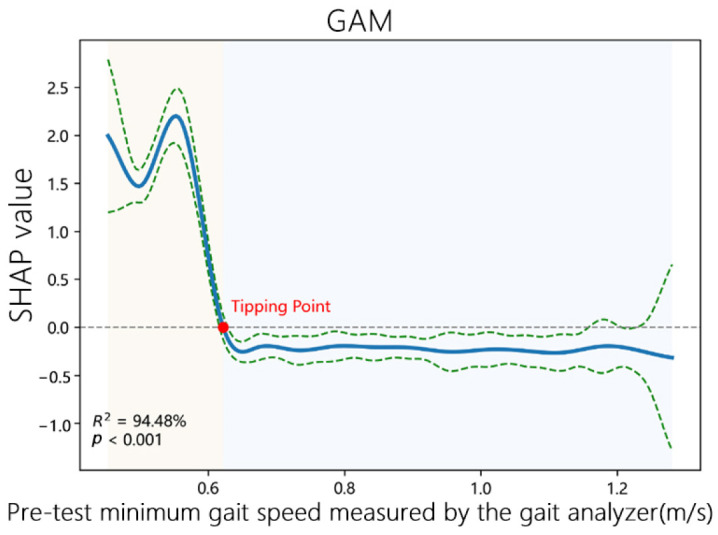
Nonlinear relationship between pre-test minimum gait speed and SHAP value.

**Figure 19:**
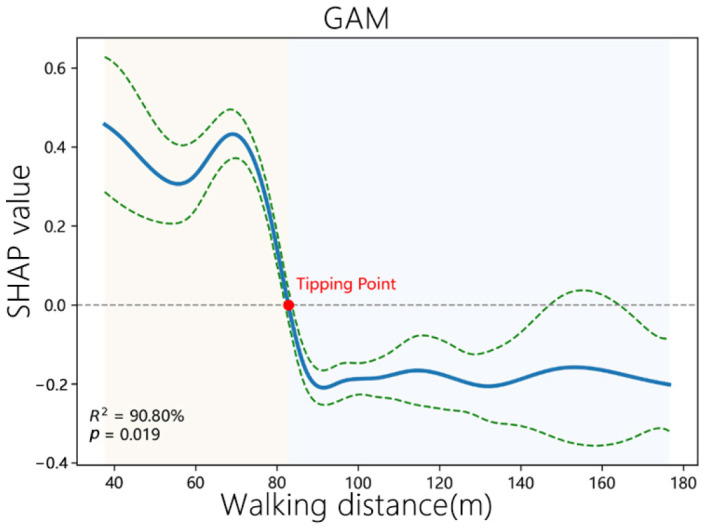
Nonlinear relationship between Walking distance and SHAP value.

**Figure 20:**
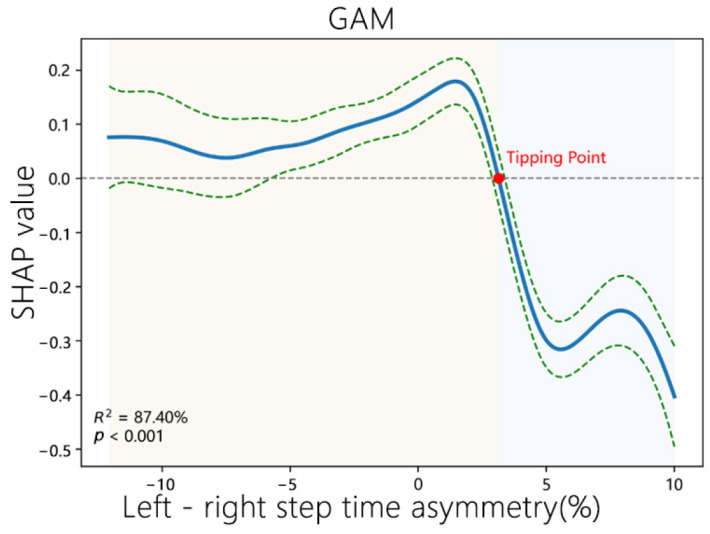
Nonlinear relationship between Left–right step time asymmetry and SHAP value.

**Figure 21:**
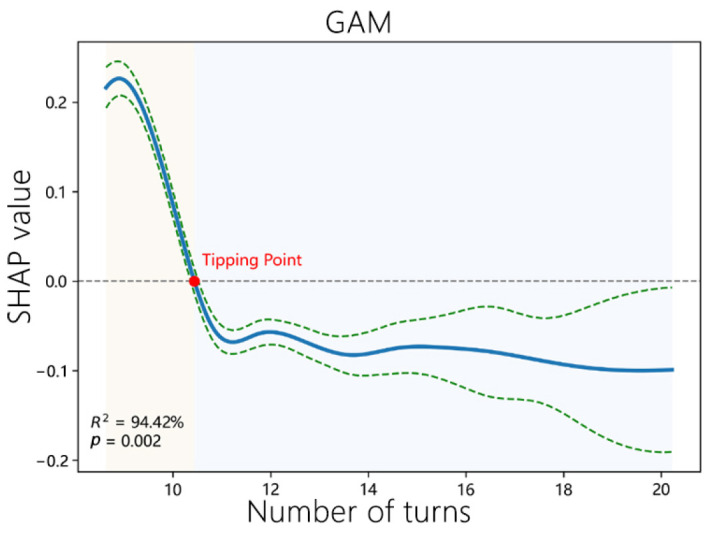
Nonlinear relationship between Number of turns and SHAP value.

**Figure 22:**
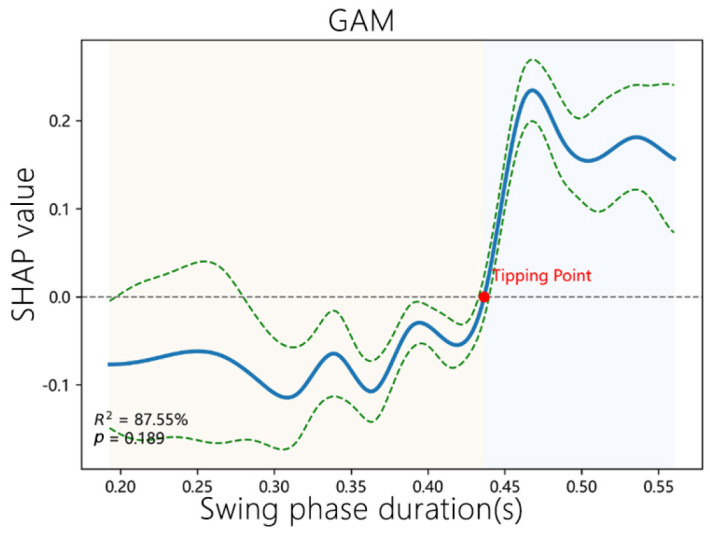
Nonlinear relationship between Swing phase duration and SHAP value.

To characterize the nonlinear contributions of key behavioral and individual features to model predictions, this study used a generalized additive model (GAM) to smooth the relationship between feature values and their SHAP values for variables selected based on SHAP importance. The turning points identified in this section are derived from the combined analysis of the random forest model, SHAP explanations, and GAM smoothing under the current sample. The purpose is to reveal nonlinear association patterns between key variables and model-predicted satisfaction. Given the limited sample size and the experimental scene with fixed acoustic beacons, a predefined route, and a specific exhibition layout, these points should not be understood as statistically validated universal design thresholds, but rather as exploratory model-explanation reference points under the current case conditions (Figure 17, Figure 18, Figure 19, Figure 20, Figure 21 and Figure 22).

For self-reported collision frequency (Figure 17), the SHAP value gradually increased as the score increased, rising rapidly before 3.25, after which the magnitude of change decreased. The curve crossed zero at approximately 2.86 (R^2^ = 98.09%, *p* < 0.001). For minimum gait speed (Figure 18), the SHAP value increased slightly around 0.49–0.56 m/s, then decreased rapidly around 0.56–0.65 m/s, crossing zero at approximately 0.62 m/s (R^2^ = 94.48%, *p* < 0.001). Walking distance (Figure 19) showed a similar trend: the SHAP value increased slightly around 56–68 m and then gradually shifted downward as the distance increased, crossing zero at approximately 82 m (R^2^ = 90.8%, *p* = 0.019). For left–right step time asymmetry (Figure 20), the SHAP value increased slightly before 1.3%, then decreased within the range of 1.3–5.4% and crossed zero at approximately 3.1% (R^2^ = 87.4%, *p* < 0.001). For the number of turns (Figure 21), the SHAP value declined rapidly around 9.6–11 turns and crossed zero at approximately 10.4 turns (R^2^ = 94.4%, *p* = 0.002). For swing-phase duration (Figure 22), the SHAP value fluctuated between 0.20 and 0.40 s and was mostly negative, then increased rapidly around 0.42–0.47 s and crossed zero at approximately 0.43 s. However, this smooth term did not reach the significance threshold and should therefore be interpreted only as an exploratory observation (R^2^ = 87.55%, *p* = 0.189).

## 5. Discussion

This study proposed and preliminarily applied an indoor accessibility assessment framework integrating multimodal sensing data and explainable machine learning. The results show that, compared with traditional research that heavily relies on participants’ post-task recall and subjective questionnaires, a multi-source data framework composed of UWB indoor positioning, gait analysis, and video-based behavioral recording can more continuously document the walking process and behavioral characteristics of people with visual impairments in a real indoor environment. Using random forest modeling and interpretability analysis, this study further attempted to link complex behavioral indicators with post-task accessibility satisfaction, providing exploratory evidence for understanding indoor mobility experience among people with visual impairments. The global interpretation results show that accessibility satisfaction among people with visual impairments was not associated with a single factor alone, but was jointly related to indicators across multiple dimensions, including safety risk, spatial cost, and motor coordination, and showed certain nonlinear patterns. This analytical pathway from multi-source behavioral data to spatial-experience interpretation provides a sensor-supported case reference for indoor accessibility assessment for people with visual impairments.

Among the above three dimensions, safety-related quantitative indicators showed the strongest explanatory contribution and exhibited obvious sensitive-response characteristics. When perceived potential danger and actual collision-related burden accumulated to a certain point, the experience could rapidly decline and then stagnate at a low level. This pattern is consistent with previous studies showing that perceived safety is an important constraint on the confidence of people with visual impairments in independent travel [13]. In addition, the distinctive contribution of this study lies in using combined sensor-supported monitoring to move the influence of safety experience from a traditionally directional and qualitative description toward data-supported quantification, thereby providing indicator-based references for subsequent spatial design rather than directly operable universal parameters.

In terms of spatial cost, this study identified nonlinear response locations for walking distance and number of turns using the sensor-supported data framework. Existing research broadly agrees that the complexity of building layouts increases the wayfinding burden for people with visual impairments [52,53], but few studies have discussed reference values for such sensitive response points. The present data show that satisfaction did not decline linearly as spatial complexity increased. The interpretability analysis suggested that before walking distance approached the sensitive range around 82.35 m, participants could still provide moderate environmental evaluations; beyond this range, the continued accumulation of path cost was associated with an accelerated decline in satisfaction. When the number of turns exceeded approximately 10.42, participants may have had to devote additional effort to route confirmation and error correction. Frequent turning not only interrupted the original wayfinding rhythm but also increased spatial cognitive load. This suggests that indoor wayfinding optimization for people with visual impairments should not simply shorten walking distance, but should also consider controlling route length and turning frequency within reasonable ranges in similar cases.

Regarding motor coordination, gait indicators captured by the photoelectric gait system, such as minimum gait speed and left–right step time asymmetry, provided a unique objective perspective in the model interpretation of satisfaction. PDP analysis showed that when minimum gait speed exceeded approximately 0.61 m/s, or when left–right step time asymmetry exceeded 3.13%, participants’ predicted satisfaction tended to decline. This indicates that gait indicators also exhibited nonlinear response characteristics in the current model. When a participant’s motor coordination was relatively disadvantaged, such as when walking speed entered a particularly low-speed or unstable range, their evaluation of spatial satisfaction could deteriorate rapidly. In clinical and human-factors research, reduced gait speed is widely regarded as a comprehensive indicator of physiological functional decline and frailty [54,55,56]. The significant contribution of gait stability in the prediction model suggests that the spatial experience of people with visual impairments is also constrained by individual motor-capacity differences. Participants with lower baseline gait ability tended to show greater sensitivity to obstacles and risks in complex indoor environments. This suggests that gait stability may serve as an observable pre-test signal for differentiated support or prior service planning in public-space management.

The multi-sensor fusion framework and experimental arrangement developed in this study provide a quantitative approach for post-occupancy evaluation of architectural-space optimization. Studies have shown that in complex indoor wayfinding tasks, a single type of sensing data often cannot comprehensively characterize user behavior; therefore, integrating multiple sensors is particularly necessary [57]. Compared with traditional indoor studies relying on smartphone positioning, the combined system used in this study—UWB positioning, multi-camera video recording, and gait analysis—has several advantages. For positioning data acquisition, smartphone positioning has limited indoor accuracy, whereas the UWB positioning system does not rely on public network signals and outputs higher-precision coordinate data through communication between its own anchors and tags, which supports more accurate walking-distance estimation. Multi-camera video recording can more accurately identify participants’ obstacle-related behaviors through complementary viewpoints, while the gait analyzer helps quickly assess participants’ motor coordination ability. At the same time, this combination of environmental-side sensing and lightweight wearable sensing has the characteristic of “one-time data collection and multidimensional feature analysis,” which can substantially reduce the economic and usability costs of long-term monitoring and behavioral research.

Based on data obtained through systematic sensor deployment and using explainable machine learning, this study identified several nonlinear response locations related to changes in experience, such as model-response regions for gait speed, number of turns, and walking distance in the current case. Compared with traditional observation, this method can more systematically present nonlinear relationships between behavioral indicators and predicted satisfaction. However, these response locations are derived from the current sample, task route, and exhibition-hall spatial conditions, and should be understood as exploratory model-explanation results rather than precise design thresholds that can be directly generalized. At the methodological level, the explainable machine-learning framework that combines objective multi-source sensing data with subjective evaluations provides a new analytical pathway for understanding indoor mobility experience among people with visual impairments, and to some extent addresses the limitations of traditional studies that rely heavily on post-task subjective interviews. Previous studies indicate that designing spaces for people with visual impairments based only on experience and intuition poses considerable challenges. The model-explanation results obtained in this study can provide preliminary references for future public-building environmental optimization and data-driven assisted design. However, these results must be further validated across more scenarios, larger samples, and different task conditions before they can become more stable design evidence.

In recent years, research on indoor navigation for people with visual impairments and accessibility in public spaces has continued to focus on the role of positioning technologies, navigation aids, and environmental design factors in supporting independent mobility. Studies from the past two years have shown that technologies such as UWB, smartphones, visual recognition, mobile robots, and multimodal interaction systems are being used to improve positioning, route following, and target-reaching abilities among blind and low-vision users in complex indoor environments [58,59,60]. Meanwhile, studies focusing on public cultural spaces such as museums have increasingly emphasized the importance of tactile interaction, clear feedback, adjustable audio prompts, and multimodal exhibition methods in supporting the independent experience of visitors with visual impairments [60]. A recent review in the field of environmental design further indicated that the wayfinding experience of people with sensory impairments depends not only on signage or assistive devices, but also on spatial layout, route continuity, environmental legibility, tactile/auditory cues, and the organization of potential obstacles [61].

Against this background, the findings of this study further suggest that the indoor accessibility experience of people with visual impairments in public cultural spaces should not be understood solely in terms of whether they can reach a target point, but also in terms of the behavioral cost and experiential quality with which they reach it. In the directed walking task conducted in the tactile museum, process-related indicators such as walking distance, number of turns, self-reported collision perception, and gait stability were all associated with predicted satisfaction. This finding indicates that even in a relatively controlled setting with acoustic beacons and a predefined route, the experience of blind users may still be jointly influenced by path burden, the complexity of node transitions, local safety perception, and physical workload. Therefore, indoor accessibility optimization for people with visual impairments should go beyond target reachability and further consider route continuity, the recognizability of key nodes, the consistency of tactile/auditory cues, and the control of hidden obstacles [61].

Compared with recent studies that have mainly focused on the development of navigation systems, the improvement of positioning accuracy, or the usability validation of assistive devices, the additional contribution of this study lies in integrating multimodal sensing data, video-based behavioral coding, and subjective evaluation to explore the relationship between behavioral characteristics during real-world mobility and accessibility satisfaction. In other words, this study does not aim to propose a new navigation device; rather, it provides a sensor-supported analytical framework for architectural space evaluation. This framework helps transform navigation outcomes from “whether users can arrive” into an experiential evaluation of “whether users can move safely, continuously, understandably, and with low burden,” thereby providing exploratory references for accessibility-oriented design and post-occupancy evaluation of public cultural buildings.

In addition, PDP analysis interprets the marginal effect of a single variable while assuming that other variables remain unchanged. However, some behavioral variables in this study may not be fully independent. For example, walking distance, number of turns, deviation, stopping, and collision/contact risk may all be influenced by the same spatial layout, route complexity, and acoustic-beacon arrangement. Therefore, PDP curves may be affected by correlated-feature bias and should not be interpreted as causal effects of independent changes in a single variable. Future studies may further introduce Accumulated Local Effects (ALE), conditional PDPs, or two-dimensional partial-dependence interaction plots to more robustly characterize nonlinear response relationships under correlated-feature conditions.

Based on the current tactile-museum case, the spatial-optimization suggestions proposed in this study should be understood as case-study-derived design implications rather than universal design standards verified across different scenarios. In the directed exhibition-viewing task set in this study, longer walking distance, more turns, and more complex node transitions were associated with lower predicted satisfaction. Therefore, for tactile exhibition halls or public cultural spaces with similar functional attributes and designed for people with visual impairments, early spatial planning may prioritize the continuity between functional nodes, clarity of route logic, and recognizability of key turning nodes. Where conditions allow, unnecessary route detours can be reduced, overly complex maze-like circulation can be avoided, tactile and auditory guidance cues can be strengthened, and hidden obstacles can be minimized to reduce path burden and safety risks during directed walking [60,61].

On this basis, spatial design and operational management should be coordinated. At the physical-environment level, potential obstacles should be controlled and sufficient clear passage width should be ensured. At key nodes where deviation or increased cognitive load is likely to occur, such as wide intersections or long open areas, tactile and auditory environmental cues should be reinforced, and property-management services and human guidance mechanisms can be integrated by setting up appropriate patrol and assistance points. Through the combination of environmental optimization and service support, a safer, more continuous, and more friendly accessible mobility environment can be created for people with visual impairments.

## 6. Conclusions, Limitations, and Future Work

This study developed an indoor accessibility assessment framework integrating multimodal sensor data and explainable machine learning. By combining gait data, indoor spatial positioning, video-based behavioral coding, and post-task subjective evaluation, this study modeled and interpreted the accessibility satisfaction of participants with first-level blindness during a directed walking task in a tactile museum. The results show that multimodal sensor data can characterize users’ spatial behavior and bodily states relatively well and can explain differences in satisfaction to a certain extent. In the current model, variables related to safety experience, route complexity, and gait stability showed relatively high predictive contributions. Some variables exhibited nonlinear associations with satisfaction and showed sensitive ranges around specific values. The contributions of this study can be divided into methodological contributions and case-based empirical findings. Methodologically, this paper proposes an analytical framework of “sensor-based data collection—machine-learning modeling—explainable analysis—sensitive-change-point identification,” enabling the translation from continuous behavioral data to contextualized spatial-design references. This provides a reusable methodological reference for data collection, variable organization, and model interpretation in other public buildings. Empirically, this study identified variables and sensitive ranges strongly associated with OAS under the current conditions of a tactile museum, fixed route, acoustic-beacon assistance, and a sample of participants with first-level blindness. Based on these results, case-specific design implications were proposed, such as reducing unnecessary turns, controlling path burden, strengthening tactile and auditory guidance, and reducing hidden obstacles. Overall, this study provides an operable technical pathway for indoor accessibility assessment based on multimodal data and explainable machine learning, and offers a methodological basis for translating continuous behavioral data into contextualized spatial-optimization references. The specific variables and value ranges still require revalidation under different buildings and task conditions.

This study has several limitations. First, the experimental scene was limited to a specific environment, and the task used a fixed-route directed wayfinding mode. In real visits, however, visitors may independently choose routes based on their interests, adjust dwell time, repeatedly visit local areas, and be affected by crowd density, environmental noise, and temporary obstacles. Therefore, the results of this study cannot be equated with behavioral performance in free visits or in other complex public-building scenarios. In addition, this study included only participants with first-level blindness and did not include individuals with low vision or partial residual vision. Differences in residual vision, age, and assistive-tool use habits may further influence behavioral characteristics and accessibility experience. The OAS, as a composite post-task evaluation indicator, covers multiple aspects, including perceived safety, route understanding, and accessible-environment experience, and may therefore obscure domain-specific effects. Some predictors in the model were subjective self-report indicators, which belong to the same subjective evaluation system as the OAS and may involve some conceptual overlap. Owing to the limited sample size, this study did not further establish independent models for each dimension, nor did it build a sensitivity model containing only objective variables. Therefore, the independent explanatory ability of objective multimodal data for the OAS still requires further verification.

Second, due to the difficulty of recruiting participants with first-level blindness and the organizational cost of real-world experiments, the sample size of this study was relatively limited, while the model input simultaneously included multiple sensor-derived indicators. Therefore, model stability may affect the stability of SHAP feature rankings and nonlinear response characteristics. Similar concerns have been reported in small biomedical and sensor-based gait datasets. For example, Trabassi et al. demonstrated in a rare-disease gait-classification context that data balancing and generative AI, combined with distributional validation and interpretability analysis, can improve classifier robustness [62]. Although the present study is not a class-imbalance classification task, this evidence highlights the need for future work to examine feature-importance stability and validate model explanations using repeated resampling or bootstrap-based strategies. The sensitive ranges and response-change points identified in this study should be understood as exploratory results based on the current sample and specific scene, rather than generalizable design thresholds.

Future research can validate the framework in more types of public buildings and with larger samples, while including participants with different levels of visual impairment, different task types, and more natural behavioral conditions to improve generalizability. It can also further compare the mechanisms influencing different experience dimensions and use repeated modeling, bootstrap validation, and feature-stability analysis to improve the robustness of model interpretations. In addition, the integration of higher-precision sensing technologies and dynamic data-collection methods may further support the transition of accessibility design from static evaluation toward dynamic response.

## Data Availability

The data presented in this study are available on request from the corresponding author. The data are not publicly available due to privacy and ethical restrictions related to the participation of blind individuals and the collection of human behavioral data.

## Supplementary Materials

The following supporting information can be downloaded at: https://www.mdpi.com/article/10.3390/s26134198/s1.

## Abbreviations

The following abbreviations are used in this manuscript:

UWB	ultra-wideband
UN CRPD	United Nations Convention on the Rights of Persons with Disabilities
UCD	user-centered design
SUS	System Usability Scale
RF	random forest
GAM	generalized additive model
POE	post-occupancy evaluation
MSE	mean squared error
SHAP	SHapley Additive exPlanations
PDPs	partial dependence plots
R^2^	the coefficient of determination

## Appendix A. List of Response Variable and Predictor Variables

**Table A1:** Response variables table.

Data Domain	Variable	Unit
Response variable	Overall Accessibility Satisfaction Score	Dimensionless

**Table A2:** Predictor variables table.

Data Domain	Variable	Unit
Basic Information/Physical Fitness Test	Sex	Categorical
Demographic and anthropometric characteristics	Age	Years
Demographic and anthropometric characteristics	Height	cm
Demographic and anthropometric characteristics	Body weight	kg
Demographic and anthropometric characteristics	Standing reach height	cm
Demographic and anthropometric characteristics	Thigh length	cm
Demographic and anthropometric characteristics	Lower-leg length	cm
Demographic and anthropometric characteristics	Upper-arm length	cm
Demographic and anthropometric characteristics	Forearm length	cm
Gait characteristics	Pre-test mean gait speed measured by the gait analyzer	m/s
Gait characteristics	Pre-test minimum gait speed measured by the gait analyzer	m/s
Gait characteristics	Pre-test maximum gait speed measured by the gait analyzer	m/s
Gait characteristics	Step length	cm
Gait characteristics	Step frequency	cm/s
Gait characteristics	Gait cycle duration	s
Gait characteristics	Stance phase percentage	%
Gait characteristics	Stance phase duration	s
Gait characteristics	Swing phase percentage	%
Gait characteristics	Swing phase duration	s
Gait characteristics	Step time	s
Gait characteristics	Left–right step time asymmetry	%
Behavioral events derived from video coding	Number of obstacle-related behaviors	Count
Behavioral events derived from video coding	Number of turns	Count
Behavioral events derived from video coding	Turning redundancy index (Tux = actual number of turns/theoretical minimum number of turns Factor)	Dimensionless
Behavioral process indicators derived from UWB positioning	Walking distance	m
Behavioral process indicators derived from UWB positioning	Walking duration	s
Behavioral process indicators derived from	Mean walking speed during the real-space task	m/s
Subjective Questionnaire	Self-reported collision frequency	Five-point rating scale
Subjective Questionnaire	Self-reported turning frequency	Five-point rating scale

**Table A3:** Questionnaire items and scoring information.

Item Code	Item Wording	Dimension	Included in OAS	Scoring Direction
Q1-exp	Can you recognize surrounding objects while moving?	Safety	No	Positive
Q1-sat	Are you satisfied with the degree of your recognition?	Safety	No	Positive
Q2-exp	Are there many obstacles in the room that interfere with walking?	Safety	No	Positive
Q3-exp	Do you feel safe while walking	Safety	No	Positive
Q3-sat	Are you satisfied with the safety of the current room?	Safety	No	Positive
Q4-exp	How often did you bump into things along the way?	Accessible environment experience	No	Positive
Q4-sat	How acceptable is the frequency of these bumps to you?	Accessible environment experience	Yes	Positive
Q5-exp	How would you rate the number of turns along the way?	Accessible environment experience	No	Positive
Q5-sat	How satisfied are you with the number of turns throughout the process?	Accessible environment experience	Yes	Positive
Q6-exp	How much difficulty did you have walking around the room alone?	Accessible environment experience	Yes	Positive
Q7-exp	When visiting the exhibition alone, how would you rate the distance between the check-in booths?	Accessible environment experience	No	Descriptive perception item
Q7-sat	How satisfied are you with the distance between the check-in booths?	Accessible environment experience	Yes	Positive
Q8-exp	How would you rate the width of the aisles inside the exhibition room?	Accessible environment experience	No	Descriptive perception item
Q8-sat	How satisfied are you with the width of the aisles inside the exhibition room?	Accessible environment experience	Yes	Positive
Q9-exp	Does the width of the indoor aisles affect your ability to find your way?	Accessible environment experience	No	Positive
Q10-exp	How would you rate the width of the corridors outside the exhibition rooms?	Accessible environment experience	No	Descriptive perception item
Q10-sat	How satisfied are you with the width of the corridors outside the exhibition rooms?	Accessible environment experience	Yes	Positive
Q11-exp	How would you rate the size of the exhibition rooms?	Accessible environment experience	No	Descriptive perception item
Q11-sat	How satisfied are you with the size of the exhibition rooms?	Accessible environment experience	Yes	Positive
Q12-exp	How easy or difficult was it to pass through the entrances of the exhibition rooms?	Wayfinding	No	Positive
Q12-sat	How satisfied are you with the width of the exhibition room entrances?	Wayfinding	No	Positive
Q13-exp	How easy or difficult was it to find the way from the entrance to the south-pointing chariot model?	Wayfinding	No	Positive
Q13-sat	How satisfied are you with the distance from the entrance to the south-pointing chariot model?	Wayfinding	No	Positive
Q14-exp	How easy or difficult was it to find the way from the south-pointing chariot to the Solar System model?	Wayfinding	No	Positive
Q14-sat	How satisfied are you with the distance from the south-pointing chariot to the Solar System model?	Wayfinding	No	Positive
Q15-exp	How easy or difficult was it to find the way from the Solar System model to the Nanhu Red Boat model?	Wayfinding	No	Positive
Q15-sat	How satisfied are you with the distance from the Solar System model to the Nanhu Red Boat model?	Wayfinding	No	Positive
Q16-exp	How easy or difficult was it to find the way from the Nanhu Red Boat model to the C919 aircraft model?	Wayfinding	No	Positive
Q16-sat	How satisfied are you with the distance from the Nanhu Red Boat model to the C919 aircraft model?	Wayfinding	No	Positive
Q17-exp	How easy or difficult was it to find the exit of the exhibition hall?	Wayfinding	No	Positive
Q17-sat	How satisfied are you with the exit guidance?	Wayfinding	No	Positive
Q18	When moving alone, which type of room do you prefer: one with simple routes and strong guidance, or one with flexible routes and minimal guidance?	Wayfinding	No	Preference item, not included in OAS

Note. (1) “Positive” means that higher scores indicate a more favorable perceived accessibility experience, including higher satisfaction, greater perceived safety, easier object recognition or wayfinding, and fewer perceived obstacles, collisions, or turns. (2) “Descriptive perception item” denotes items that describe perceived spatial attributes, such as distance, corridor width, or room size. (3) “Included in OAS” indicates whether the item was used to calculate the Overall Accessibility Satisfaction Score. All items included in the OAS were coded in the same evaluative direction before aggregation, with higher total scores representing higher overall accessibility satisfaction. (4) The Overall Accessibility Satisfaction Score (OAS) was calculated by summing the scores of the seven included items, each rated on a five-point Likert-type scale. Therefore, the theoretical total score ranged from 7 to 35, with higher scores indicating greater OAS for the experimental space. (5) The route-preference item comparing strong guidance with high route freedom was excluded from the OAS calculation.
