# Supplementary material for: An Indoor Accessibility Assessment Framework Based on Multimodal Sensing and Explainable Machine Learning: A Case Study of a Tactile Museum for People with Visual Impairments
Source: Sensors (Basel). 2026 Jul 2;26(13):4198. doi: 10.3390/s26134198 (PMC13364338; doi:10.3390/s26134198)
Supplement: Supplementary file 1 [file sensors-26-04198-s001.zip › sensors-4353112-supplementary.pdf]

Reliability and item-total statistics of the OAS.

OAS item	CITC
Q4_sat	0.312
Q5_sat	0.334
Q6_exp	0.196
Q7_sat	0.098
Q8_sat	0.361
Q10_sat	0.241
Q11_sat	0.291

Note：

CITC = Corrected Item-Total Correlation. All CITC values were positive, indicating generally consistent scoring directions, although several items showed weak item-total associations. The seven-item OAS had Cronbach's $\alpha = 0.525$ and McDonald's $\omega = 0.709$.
